# Carbon Material and Electrolyte Composition Effects on the Performance of Prussian White Solid Boosters in Flow Batteries

**DOI:** 10.1021/acsomega.6c04178

**Published:** 2026-07-24

**Authors:** Vahid Abbasi, Eduardo Martínez-González, Rosa Tirronen, Ayesha Kousar, Eeva-Leena Rautama, Pekka Peljo

**Affiliations:** † Department of Chemistry and Materials Science, Aalto University, 00076 Aalto, Finland; ‡ Mechanical and Materials Engineering Department, University of Turku, 20014 Turku, Finland

## Abstract

Prussian White-based boosters were prepared using four commercially available carbon materials as conductive additives and cycled at the storage tanks of flow batteries with ferri/ferrocyanide-based electrolytes. All systems underwent a 21–22 day charge–discharge cell test to assess their long-term cycling stability; boosters containing the carbon material YP-50F completed nearly twice as many cycles as the other studied carbons (e.g., 500 vs 194, for YP-50F and C65, respectively), exhibiting their faster charge–discharge behavior. Changing the carbon material had a slight effect on the capacity utilization of the solid boosters (variations ∼10% under similar conditions), and this trend persisted across all remaining experiments. A significant improvement in capacity was observed when increasing the ferri/ferrocyanide concentration from 0.1 mol L^–1^ to 0.8 mol L^–1^ in the electrolyte solution, with the maximum capacity utilization increasing from 22.1% to 94.8% for a booster made with YP-50F carbon material. The results suggest that the increased booster utilization capacity observed at higher electrolyte concentrations is related to an enhanced mass transfer through the particles and a better matching between the redox potential of the boosters and the electrolyte.

## Graphical abstract

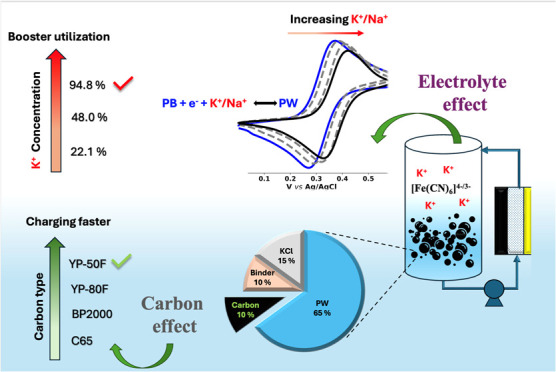

## Introduction

The transition to renewable energy sources (e.g., sun and wind) is creating an increasing demand for developing advanced, robust, and scalable energy storage technologies. Flow batteries (FBs) are a flexible technology for stationary and large-scale energy storage; they decouple power (set by cell design) and energy density (set by storage tank size) by storing energy in liquid electrolytes in external tanks while providing high safety and extended cycle life.^1–5^ Despite these advantages, FBs remain considerably less competitive than Li-ion batteries in terms of energy density, positioning the development of high-energy-density storage systems as a key priority to accelerate flow battery (FB) improvements, especially for vanadium-free systems. While designing aqueous FBs with high-energy density is a promising approach to address this problem, it remains challenging for the following reasons.^6–11^

The energy density of FBs is limited by solubility and/or faster degradation of electroactive materials at high concentrations.^12–16^ Electrolyte development is progressing rapidly for FB negolytes (solution to store negative charge), whereas improvements in posolyte materials are limited, which also imposes restrictions on cell voltage modulation.^17–21^ An alternative strategy to enhancing energy storage relies on incorporating solid boosters (pellets mainly composed of an insoluble electroactive material with a redox potential that matches closely that of the water-soluble electroactive species) at the storage tanks.^22–24^ In battery charging mode, the soluble redox couple acts as a charge-transporting species, taking charge from the electrode surface and further transferring it to the boosters at the reservoir. The opposite charge-transfer process occurs when discharging the battery. Thus, a synergistic contribution between boosters and electrolytes to energy storage arises, which is key to developing compact systems of enhanced energy density. In other words, the solubility of the electroactive material is no longer critical for energy storage density, as most of the energy can be stored in the solid boosters. This FB configuration is promising for advancing the performance of the scarce posolytes that have already been reported.^21,25^

One of the most widely tested posolytes utilizes cost-effective ferri/ferrocyanide species ([Fe­(CN)_6_]^3–/4–^) as the electroactive material; both species are air-stable and can operate in neutral solutions at a relatively high redox potential (0.35–0.46 V vs SHE depending on the electrolyte and concentration^26,27^ ), without experiencing significant material degradation over multiple charge–discharge cell cycles (5000 cycles)^28^ in neutral-alkaline pHs.^29–31^ Nevertheless, the redox couple [Fe­(CN)_6_]^3–/4–^ undergoes a single-electron transfer process (eq 1) and has relatively low water solubility, 1.6 M [Fe­(CN)_6_]^4–^ at maximum by a equimolar mixture of K_4_[Fe­(CN)_6_] and Na_4_[Fe­(CN)_6_] in 1.0 M KCl.^29^ Due to these limitations, ferri/ferrocyanide-based electrolytes are typically required in volumetric excess to support the operation of high-performing or high-capacity negolytes.^32–34^

1 $${\mathrm{Fe}\left(\mathrm{CN}\right)}_{6}^{3-}+e^{-}⇌{\mathrm{Fe}\left(\mathrm{CN}\right)}_{6}^{4-}$$

Leveraging the low water solubility (far below 0.001 mol L^–1^ in KCl), and the reversible one-electron transfer process (eq 2) by the redox couple Prussian Blue (PB)/Prussian White (PW) at 0.33–0.46 V vs SHE, PW-based boosters can be prepared and tested as redox-active materials into the storage tank of a neutral ferri/ferrocyanide-based posolyte.^35–38^ When oxidizing PW at the storage tank, the Fe^2+^ species within its Fe^2+^–N≡C–Fe^2+^ linkages lose electrons to form Fe^3+^ species, while K^+^/Na^+^ ions are deintercalated from the lattice.^37,39,40^ Pioneering works on this system,^41–43^ which also served to demonstrate the concept of storage tanks with enhanced energy density, reported FBs with exceptional long-term cycling stabilities over 7000 charge–discharge cell cycles and few details regarding the preparation methods and formulations of the boosters.^38^

2 $$\begin{array}{l} X^{+}+{\mathrm{NaFe}}^{3+}\left[{\mathrm{Fe}}^{2+}{\left(\mathrm{CN}\right)}_{6}\right]+e^{-}⇌{\mathrm{XNaFe}}^{2+}{\left(\mathrm{CN}\right)}_{6}]\\ X^{+}=K^{+}/{\mathrm{Na}}^{+} \end{array}$$

Prussian Blue analogues are well-established electrode materials with an open three-dimensional framework that accommodates alkali ions reversibly.^44^ [Fe­(CN)_6_] vacancies and crystal water are the main sources of structural degradation during cycling, driving side reactions and capacity fade. Recent work has addressed these issues through metal substitution,^45^ reductive synthesis,^46^ and morphological control,^47^ consistently demonstrating that lower vacancy density and water content translate to improved cycling stability.

Based on the literature,^25,48–50^ binder and carbon materials as conductive additives in the boosters’ composition are essential to preserve structural integrity and electrical conductivity; otherwise, the particles rapidly disintegrate, releasing solid material into the cell, blocking the flow. Carbon and binder materials are also important to tailor enhanced electrodes for Li-ion batteries and capacitors, where it has been found that varying the properties of these materials not only affects the long-term cycling stability of the resulted electrodes but also their counterion intercalation process and energy storage capacity.^51–56^ Therefore, it is promising to study how these materials influence the performance of PB- and PW-based boosters incorporated at the storage tank of a ferri/ferrocyanide-based electrolyte, paying significant attention to reaching a good capacity utilization and long-term cycling integrity of the particles.

Building on these promising directions, Rourke et al.,^57^ studied three different carbon black materials and added KCl as dissolvable pore forming agent to the formulation of PB-based boosters, showing significant effects of material composition on the capacity utilization, rationalized as a function of the particles’ porosity, where small amounts (around 10–15%) of KCl were found to be optimal. In a separate study,^58^ the authors also suggested that capacity utilization depends on the ferri/ferrocyanide concentration and the ions (K^+^, Na^+^, Li^+^) present in the charge-transporting solution. Despite this, more experimental information is needed to gain further insight into the phenomenon, as even after optimizing the formulation and FB electrolyte, the booster particles yielded capacity utilization values of 35%^58^ and 46%,^57^ which are clearly lower than 57.6% and 61.0%, reported by Chen et al.^59^ and Yan et al.,^38^ respectively. Therefore, it is promising to study these effects not only to improve the performance of PB- or PW-based boosters but also to elucidate fundamental principles that could support the development of boosters for other FB electrolytes.

Following this approach, we prepared PW-based boosters using formulations containing 15% KCl, the binder PEAA poly­(ethylene-*co*-acrylic acid), and four different carbon materials, three of them not studied for this application before. By charging–discharging the particles with FB electrolytes containing a mixture of Na^+^ and K^+^ ions and different ferri/ferrocyanide concentrations, the aim was to get more clues on how these materials affect not only the FB performance and capacity utilization but also the long-term cycling integrity of the particles. The results showed modest carbon material effects on the boosters’ capacity utilization, but a significant impact on both the cell discharging times and the long-term cycling integrity of the particles. Mixing counterions allowed us to examine electrolyte effects at higher concentrations of the water-soluble electroactive species than those utilized by Rourke et al.,^57^ which was crucial to maximize the capacity utilization of the boosters. By optimizing the boosters’ formulation and electrolyte composition, we demonstrated that our boosters can sustain long-term cycling integrity (over 500 charge–discharge cell cycles) and achieve high-capacity utilization of up to 94.8%. Cyclic voltammetry experiments revealed that redox matching between both electroactive materials is affected by the concentration of K^+^ species in solution, which shifts the redox potential of PB/PW. As the changes in the redox matching are rather small, it is proposed that improvements in booster utilization with increasing concentrations are due to improved mass transfer between solids, especially inside the beads and liquid. Therefore, for future work, finding a proper concentration and mixture ratio of species K^+^ and Na^+^ in the charge-transporting electrolyte will be crucial to maintain a good ferri/ferro water solubility, while enhancing the boostes’ performance.

## Experimental Section

### Boosters’ Preparation

Materials PW, KCl, and carbons, along with the binder poly­(ethylene-*co*-acrylic acid) (PEAA), were commercially available. The carbon black (CB) material was varied in the formulation, and each resulting batch of boosters was identified by its corresponding carbon code. The process began by weighing and mixing desirable mass ratios of powders PW (6.5 g) and CB (1 g). Separately, 1 g of PEAA and 1.5 g of KCl were added to 20 mL of THF solvent. Once PEAA fully dissolved, the solution was poured into 7.5 g of the PW-CB mixture, and the resulting mixture was stirred for around 5 min. Then, the formed paste was left in the fume hood to partially evaporate THF until a viscous paste of desirable consistency was obtained. The partially dried paste was then loaded into an extruder (having 3 mm diameter holes) to shape the boosters by a single compression lasting about 5 s. The resulting particles were left to dry overnight. All steps were carried out in a fume hood.

### Battery Tests

Battery cycling tests were performed using a LANHE Battery Cycler G340A (LANHE, China), circulating the electrolytes with a BT600 M peristaltic pump (Baoding Chuangrui, China) at a flow rate of 35 mL/min. Three cell sizes were employed, with larger cells used for higher-concentration electrolytes; the active membrane areas were 5, 9, and 20 cm^2^. All cells utilized a carbon felt as the working electrode. A Nafion 212 cation-exchange membrane was sandwiched in the middle of the cell with Teflon gaskets to separate the positive and negative electrolytes.

### Cyclic Voltammetry

Cyclic voltammograms were recorded using a PalmSens4 potentiostat (PalmSens, Netherlands), with a glassy carbon working electrode (*d* = 2 mm), a platinum wire counter electrode (*d* = 0.5 mm), and an Ag/AgCl (3 mol L^–1^ KCl) reference electrode.

### Chemicals

All chemicals were used without further purification unless otherwise stated. Carbon materials included YP-50F and YP-80F (Kuraray Chemicals), C65 (Nanografi), and BP2000 (Cabot). Prussian White was obtained from Altris (Sweden), while poly­(ethylene-*co*-acrylic acid) (PEAA), KCl, and NaCl were sourced from Sigma-Aldrich. All solutions were prepared using ultrapure water (18.2 MΩ·cm, Millipore Direct-Q 5 UV). The membrane was purchased from Ion-power, USA. Thermally activated carbon felts GFD 4.6 mm from SIGRACELL as an electrode for battery tests. Sodiated PW was sold with the brand name of Fennac by Altris.

## Result and Discussion

### Materials Characterization

The X-ray diffraction (XRD) and scanning electron microscopy (SEM) results for the Prussian White material are shown in Figure 1. Prussian White typically consists of cube-shaped particles. As the compound was stored at ambient conditions, the SEM images (Figure 1, bottom) show a significant loss in the original morphology, with particles of ∼10 μm size. The original cubic morphology is mostly preserved but significantly altered, exhibiting much rougher surface texture compared to the fresh material.

**Figure 1:**
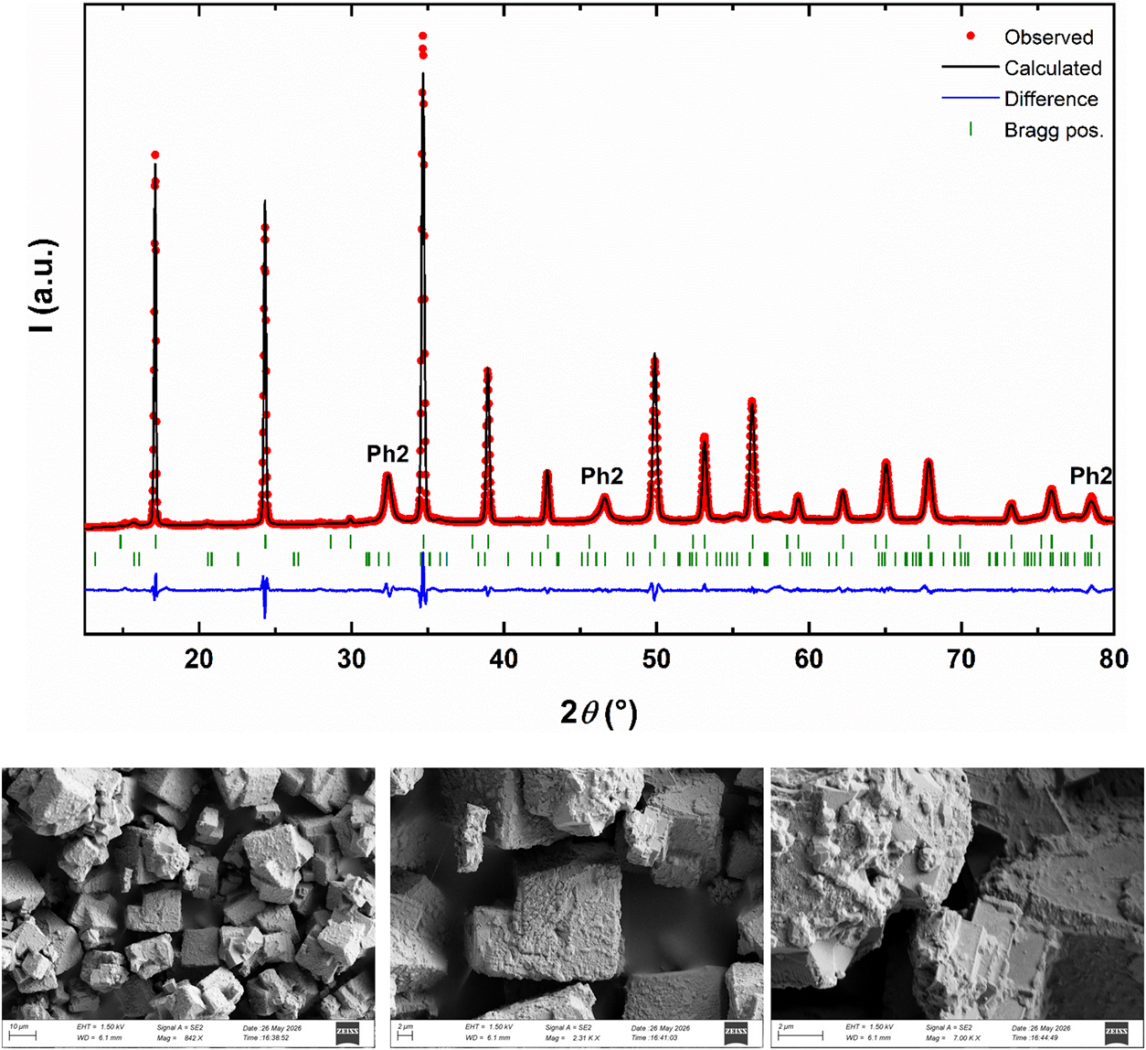
SEM images (bottom) and Le Bail fitting of the XRD pattern (top) from the PW active material after 6 years of storage at ambient conditions. The data are shown in the variable divergence-slit (VDS) mode instead of the standard fixed-divergence one (FDS). The top row of bars represents the cubic phase (a ≈10.33 Å), and the bottom row of bars all possible Bragg peaks in general *Pmmm* symmetry in the used lattice dimensions. The main Bragg reflections not explained by the cubic phase, also broader in their peak shapes, are marked as Ph2. One should note that the last reflection received no intensity from the cubic model (VDS or FDS).

In terms of composition, XRD (Figure 1, top) revealed the material consists of two sodium-rich phases: from a well-known cubic main phase (*Fm*3̅*m*) with a fitted lattice parameter of *a* = 10.3312(2) Å and a secondary phase, for which a match was found only with a poorly characterized reference (ICDD #00–001–1026, Na_4_Fe­(CN)_6_). The obtained value of the lattice parameter suggests the main phase composition lacks Na^+^ compared to its well-known analogue, assigned to Na_0.52_Fe­[Fe­(CN)_6_] [ICDD #01–083–5999; *a* = 10.36 Å], but is not Na-deficient (*a* = 10.23 Å) either.^60^ A simple interpolation, typically applicable, suggests a composition Na_0.35_Fe­[Fe­(CN)_6_] for the present sample.

Based on the obtained indexing results for the unknown secondary phase, the general orthorhombic space group *Pmmm* was applied in the total pattern fitting. A reasonably good match was obtained with a refined lattice of *a* = 6.718(1) Å, *b* = 5.518(1) Å, and *c* = 5.615(2) Å, albeit one cannot rule out a monoclinic distortion with the current phase formation. It is also worthwhile to mention that, as the used fitting software allows it, the fitting was made in a variable slit mode to maintain the overcounted intensities at the high angles to add visual accuracy to the model.

Since the crystal structure of the secondary phase remains unknown, its chemical composition and quantification can only be speculated. Na_3_Fe­(CN)_6_ is a plausible option considering that Fe^2+^ in the original reference is an unlikely form after oxidizing conditions. Having an orthorhombic (or a monoclinic) symmetry in a cubic main matrix, its relative intensity would suggest a maximum 10% phase interaction.

Based on these results, having ∼90% Na_0.4_Fe­[Fe­(CN)_6_] and ∼10% Na_4_[Fe­(CN)_6_], Na_0.4_Fe­[Fe­(CN)_6_] storage capacity is considered to be one electron with 96.9 mAh/gr of electroactive material, and Na_4_[Fe­(CN)_6_] capacity resulted to be 88.1 mAh. Then, for estimating the theoretical booster’s capacity in all the cases, 96.0 mAh/gr of PW was considered.

With respect to carbon materials, their SEM images (Figure S1) show that Super C65 consists of rounded, loosely aggregated particles with smooth surfaces, while BP2000 forms much finer, tightly packed agglomerates with a more complex aggregate structure. In contrast, YP-50F and YP-80F exhibit a dense sheet-like morphology with rough surfaces and visible micropores. Raman spectra (Figure S2) also show highly disordered carbon materials YP-80F, YP-50F, Super C65, and BP2000, as evidenced by their broad D and G bands, *I*_D_/*I*_G_ ratios clustered between 1.03 and 1.18, and weak suppressed 2D bands around 2700 cm^–1^. These features collectively confirm the absence of significant long-range graphitic order in any of the four carbons. Overall, the four materials are similar from a structural standpoint, and any performance differences between them in application are likely governed more by surface area, porosity, and particle morphology than by graphitic order alone. Some characteristics of these carbon materials are listed in Table 1.

**Table 1:** Main Characteristics of the Four Commercially Available Carbons Used as Conductive Materials

Carbon material	Bulk density (g cm^–3^) at 20 °C	Skeletal density (g cm^–3^)	Surface area (m^2^ g^–1^)	Pore width (nm)	Morphology	Refs
BP2000	0.2–0.38	1.7–1.9	1500	0.7–35 avg 14.7	Tight, cauliflower-like agglomerates	^61,62^
YP-50F	0.30	0.68	1692	0.5–7 avg 2.26	Sheet-like structure with scattered micropores	^61,63,64^
YP-80F	0.18	0.22	2271	0.5–3	Sheet-like structure with scattered micropores	^63,65^
C65	0.16	1.6	62	5–70	Rounded, loosely aggregated particles	^66,67^

### Long-Term Cycling Stability

Pioneering works demonstrated the long-term cycling stability of FBs containing PB-based boosters.^38,59^ Boosters’ performance is significantly affected by their formulation.^22,57,58^ As for this work, we varied the carbon material of the PW-based boosters, which could affect their structural integrity; the long-term cycling stability for each batch of boosters obtained was initially tested. The percentages of materials in the tested boosters are 65% PW, 10% CB, 15% KCl, and 10% binder. All batches of boosters were tested in the posolyte tanks of symmetric FBs, placing ferro/ferricyanide-KCl solutions with excess capacity in the negolyte tanks.

Starting with the already studied carbon black C65,^57^ its boosters were tested with 30 mL of an electrolyte containing 0.1 mol L^–1^ ferricyanide in 1 mol L^–1^ KCl. A fast charging–discharging process with constant current (CC) density values of ±40 mA cm^–2^ was applied to the cell, aiming to provide a reference point for the systems’ capacity and performance, from which optimization was initiated. In all the experiments, ferricyanide species were placed in the posolyte tank to trigger immediate charging of the boosters, so the first cycles were used to prewet the particles, placing enough ferri/ferrocyanide in the negolyte tank to completely discharge the particles in subsequent cycles. In the absence of solid boosters (Figure 2A, red line), the solution retained 92.6% of the theoretical capacity (80.4 mAh). When increasing the tank capacity by 247.5 mAh through the addition of the boosters (Figure 2A, blue color), the system only utilized 11.3% of this capacity, which may be due to slow electron transfer kinetics at the boosters-electrolyte interface, probably due to limited K^+^ intercalation at the PW lattice.^35,39,40^ This kinetic limitation becomes more pronounced when increasing the current density (Figure 2B); slow charging–discharging processes are needed to access higher capacity utilization of the boosters.

**Figure 2:**
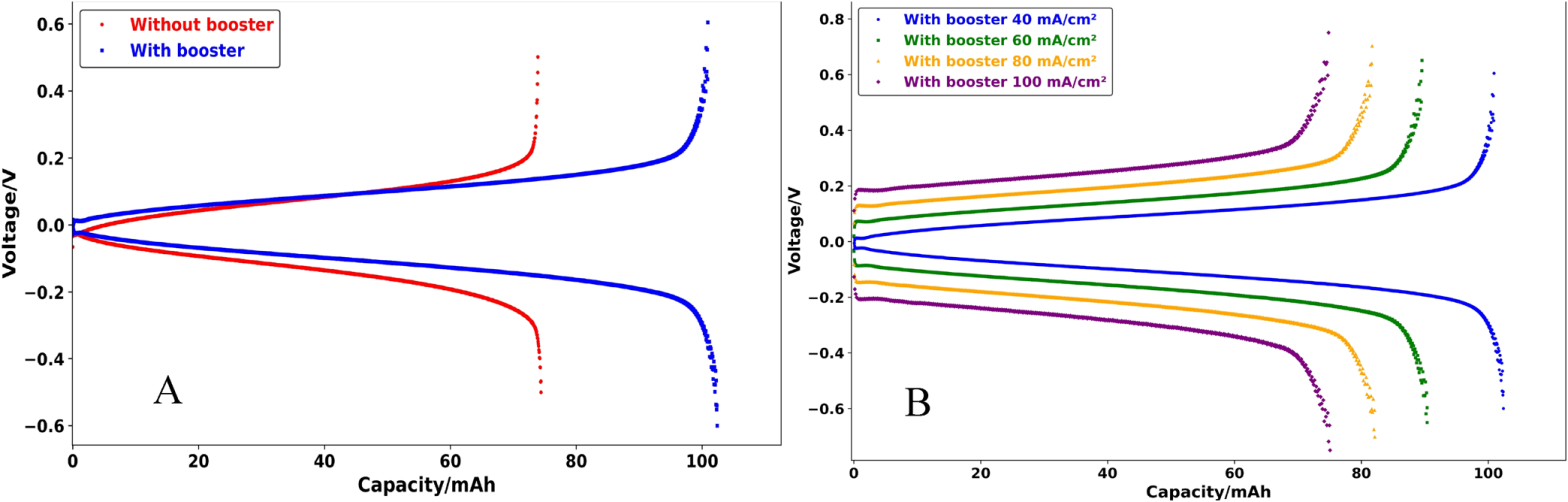
Charge/discharge profile of symmetrical cell without booster in red and with booster in blue profile 40 mA/cm^2^ (A) charging booster in different current densities (B). 30 mL of 0.1 mol L^–1^ ferricyanide in 1 mol L^–1^ KCl is used as the posolyte (+0.00922 mol PW in C65 booster with capacity of 247.5 mAh), and 0.1 mol L^–1^ ferrocyanide +0.01 mol L^–1^ ferricyanide in 1 mol L^–1^ KCl as the negolyte in excess volume. The experiment was conducted in a cell with a 5 cm^2^ membrane area.

To optimize time, fast CC followed by constant voltage (CV) was applied, because while reducing CC could be useful, the optimal value of current density is unknown. Fast CC rapidly advances most of the reaction, and CV allows it to gradually reach the maximum capacity utilization of the boosters, under the conditions tested. For the CC step, ±60 mA cm^–2^ and ±0.6 V cutoff values were chosen, while the CV step consisted of applying ±0.6 V with cutoff current values of ±6 mA cm^–2^. Although these conditions may remain insufficient to access the maximum capacity utilization of the booster, particularly due to the ±6 mA cm^–2^ limits imposed at the CV step, the global protocol was appropriate to evaluate the long-term cycling integrity of the boosters during more than 19 days. This procedure was applied for all the studied systems, considering a similar electrolyte composition and a theoretical booster capacity of 247.5 mAh, as in the previous test. The discharge capacity of the boosters containing different carbon materials is plotted as a function of the number of cycles in Figure 3, indicating the capacity of the solution by an arrow, while their percent of capacity utilization and FB cycle time duration are compiled in Table 2.

**Figure 3:**
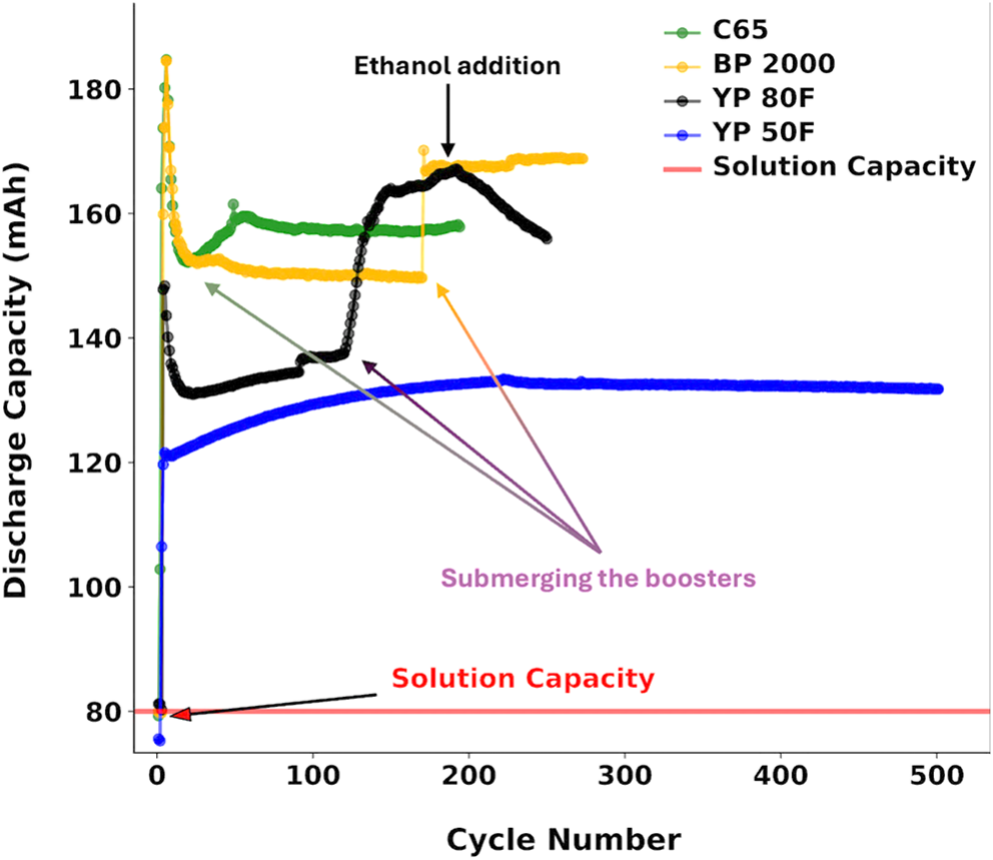
Comparison of boosters’ utilization capacity with different carbon materials in the CC–CV process mode. The posolyte tanks contain 30 mL of 0.1 mol L^–1^ ferricyanide in 1 mol L^–1^ KCl + booster with a capacity of 247.5 mAh. The solutions were paired with 80 mL of negolyte solution containing 0.1 mol L^–1^ ferrocyanide and 0.01 mol L^–1^ ferricyanide in 1 mol L^–1^ KCl.

**Table 2:** Details from Batteries Tested with CC–CV Protocol, Capacity Utilization of the Booster Means the Discharged Capacity from Booster Divided by 247.5 mAh (Theoretical Capacity of Booster Containing 0.00922 mol PW)

Carbon material used in booster	Capacity utilization (%)	One full cycle duration (hr:min)	Estimated time to 100%
BP2000	35.9	1:31	4:13
YP-80F	35.3	2:21	6:39
C65	32.2	2:23	7:24
YP-50F	22.1	0:56	4:13

Systems based on the carbon materials YP-80F (from cycle 120) and BP2000 (from cycle 190) exhibit a sudden increase in capacity; this is attributed to an initial floating of the booster beads; the beads were not wetted, resulting in poor contact with the solution until we fully immersed them and placed glass wool on top as additional support. In the case of YP-80F, we tried to modulate the redox potential of electroactive species through alcohol–water mixtures, as suggested by Liang et al.^68^ However, pouring 1 mL of ethanol (after cycle 194) into the boosted tank caused a rapid, continued loss of storage capacity continuing with each subsequent cycle; therefore, this test was aborted and not applied to the rest of the systems.

Although significant improvement was observed with the CC–CV protocol, resulting in moderately higher capacity utilization than previously reported for carbon C65 (16% for 0.1 mol L^–1^ ferrocyanide),^58^ all boosters continued to exhibit low capacity utilization: 35.9% (88.9 mAh), 35.3% (87.3 mAh), 32.2% (79.6 mAh), and 22.1% (54.8 mAh) for BP2000, YP-80F, C65, and YP-50F systems, respectively. These variations could be related to changes in porosity induced by the different carbon materials and the addition of KCl to the formulation.^57^ However, the variations and maximum booster capacity utilization were found to be less than 10% and 28%, respectively; so, optimizing these properties does not appear to be the way to further improve the capacity of the particles.

The long-term cycling stability for all batteries studied was demonstrated in cells running 21–22 days (nearly the same duration), where the system operated with carbon material YP-50F showed outstanding behavior, exhibiting a significantly larger amount of charge–discharge cell cycles (e.g., 500 vs 194, for YP-50F and C65, respectively, Figure 3) than the remaining systems, suggesting a significant impact of the carbon material on the charging–discharging rate of the boosters, although YP-50F showed the lowest capacity utilization.

### Carbon Material Effects on FB Discharging Times

Carbon material effects on the charging–discharging times are evident from the results presented above (Table 2). To examine this in more detail, and with the intention of studying systems operating with higher capacity utilization, a combined (fast) CC–(slow) CC process was employed over more than 40 cycles and the results are presented in Figure 4, imposing ±72 mA cm^–2^ and ±3 mA cm^–2^ to the cell, successively with the ±0.55 V cutoff. The boosted tanks contained a theoretical capacity of 331.7 mAh, with 144.1 mAh capacity provided by the boosters and 187.6 mAh from the solution in the posolyte tank. For these experiments, the ferricyanide concentration in the charge-transporting (20 mL) solution was increased to 0.35 mol L^–1^, and a mixture of 0.5 mol L^–1^ NaCl with 0.5 mol L^–1^ KCl was used as a supporting electrolyte. The mixture of counterions is relevant when increasing the concentration of the water-soluble electroactive material,^69^ while maintaining good intercalation of counterions at the booster particles when cycling.

**Figure 4:**
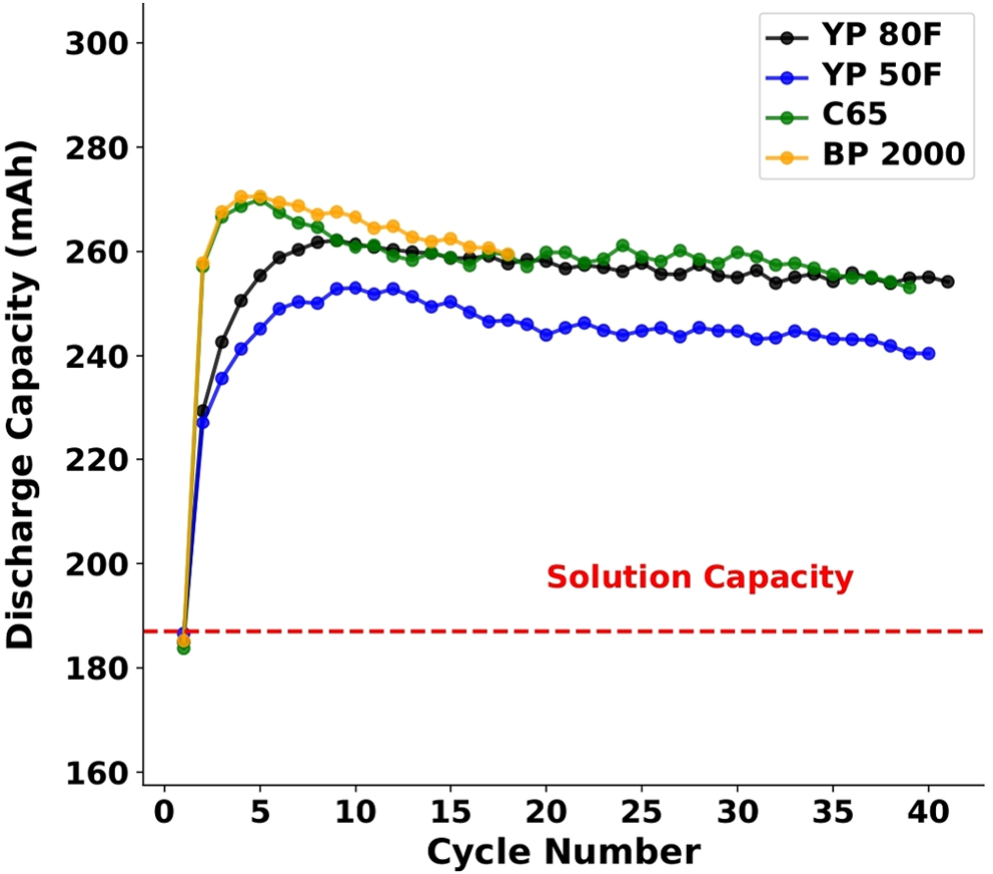
Comparison of booster utilization for different carbon materials under CC (fast, 72 mA cm^–2^)–CC (slow, 3 mA cm^–2^) process mode with ±0.55 V cutoff. 20 mL of 0.35 mol L^–1^ ferricyanide in 0.5 mol L^–1^ NaCl and 0.5 mol L^–1^ KCl as electrolyte (187.6 mAh) + boosters (144.1 mAh) placed as posolyte. 0.35 mol L^–1^ ferrocyanide +0.035 mol L^–1^ ferricyanide, and the same supporting electrolyte was placed in excess volume as the negolyte.

It was not possible to complete the test for the boosters containing carbon material BP2000, since this system underwent fast particle disintegration causing booster material to enter the cell, blocking the flow and damaging the pipe. This experiment was repeated three times and, in each case, the booster material dissolved. This result shows that the carbon material properties also affect the long-term mechanical stability of the boosters. Overall, the systems demonstrated Coulombic efficiencies above 99.5%, indicating excellent charge recovery and highly reversible electrochemical processes. A significant improvement in the percentage of capacity utilization was achieved with the CC–CC protocol and experimental conditions. The resulting values for the boosters containing carbon materials BP2000, YP-80F, YP-50F (Figure 4), and C65 were 59.9%, 54.0%, 48.0%, and 59.7%, respectively. Although slowly applying ±3 mA cm^–2^ to the cell likely resulted in more time for the particles to be charged-discharged, it should also be noted that ferricyanide concentration was increased in this test, which has been reported to further improve the capacity utilization of PB-based boosters.^58^ Consequently, the cycle time duration for all systems was analyzed, where data revealed (Table 3) a trade-off between capacity utilization and cycle duration among the four tested carbon materials. A similar result can be detected from Table 2.

**Table 3:** Details from Batteries Tested with CC–CC Protocol, 144.1 mAh (Theoretical Capacity of Booster Containing 0.005376 mol PW)

Carbon material	Capacity utilization (%)	One full cycle duration (hr: min)	Estimated time for one cycle to 100% capacity
BP2000	59.9	11:06	18:31
C65	59.7	7:06	11:54
YP-80F	54.0	8:18	15:22
YP-50F	48.0	6:42	13:57

Boosters containing BP2000 achieved the highest capacity utilization at 59.9%, but this was accompanied by the longest cycle duration of 11:06 h, suggesting slower kinetics or charge-transfer limitations. In contrast, the system containing C65 delivered a comparable capacity (59.7%) with a significantly shorter cycle duration of 7:06 h, reflecting a more efficient balance between energy storage and cycling speed. YP-80F exhibited moderate performance, with a capacity utilization of 54.0% and a cycle duration of 8:18 h, while YP-50F demonstrated the fastest cycling at 6:42 h but the lowest capacity utilization at 48.0%. C65 provides the most favorable balance between capacity and cycle time, although its cycling stability remains a concern.

For future work, suitable binders preserving integrity could be explored for this system and for the remaining materials, as suggested by Sepp et al.^25^ Overall, these results clearly highlight a significant impact of carbon material properties on the charge–discharge cycle duration of the boosters. With an appropriate selection of carbon materials, faster cycling can be achieved with minimal capacity loss, which is critical for advancing booster technology toward practical applications.

From the above-discussed experiments, it is evident that slow charging (3 mA cm^–2^) is necessary to significantly increase the capacity utilization of the boosters, which may be due to a slow electron transfer kinetics at the boosters–solution interface.^21–23^ Then, current density capability tests were performed for the three systems that did not disintegrate, applying different current density values (during 10 cycles for each current density) in the following order: ±72, ±60, ±48, ±36, ±24, ±12, and ±6 mA cm^–2^. The percentage of booster utilization capacities is presented in Figure 5.

**Figure 5:**
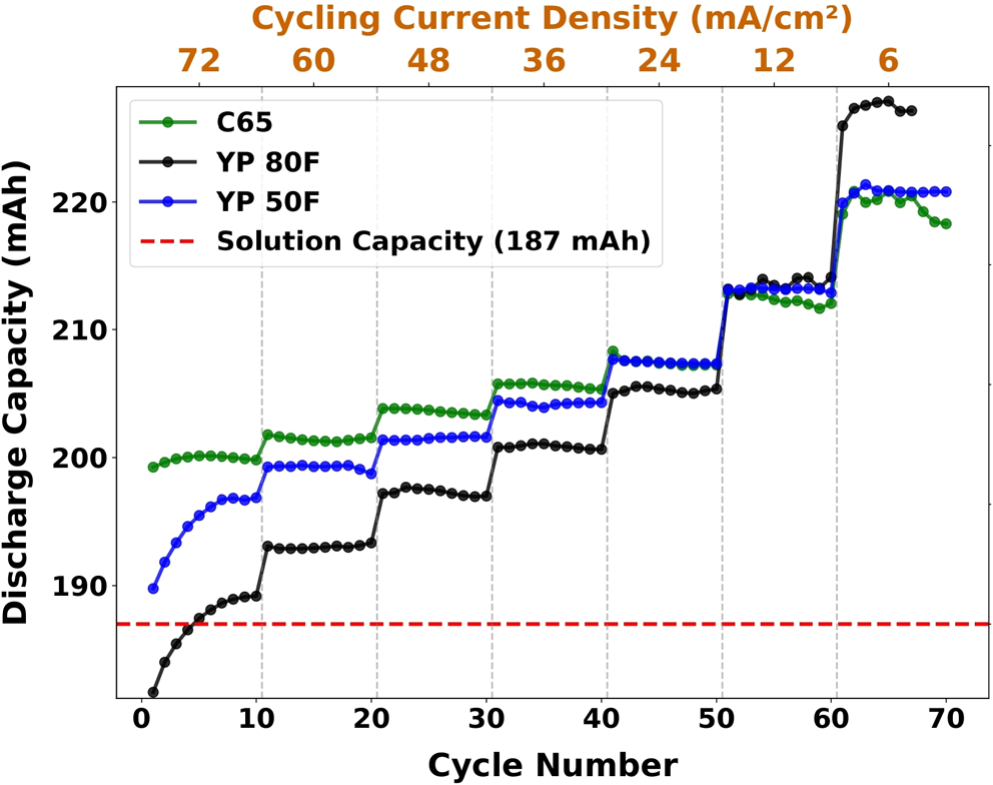
Comparison of boosters in different charging rates in the CC process with ±0.5 V cutoff. 20 mL of 0.35 mol L^–1^ ferricyanide in 0.5 mol L^–1^ NaCl and 0.5 mol L^–1^ KCl was placed as the posolyte (187.6 mAh), adding boosters (144.1 mAh) in the tank. 0.35 mol L^–1^ ferrocyanide +0.035 mol L^–1^ ferricyanide was placed in excess volume in the same supporting electrolyte as the negolyte.

At the end of the test, the battery containing C65-based boosters exhibited leakage due to material disintegration, showing limited promise for long-term use, at least under the experimental conditions and formulations tested here. This result reinforces the proposal that carbon material properties strongly impact the long-term cycling stability of the studied boosters.

As expected, Figure 5/Table 4 demonstrates that decreasing the current density in all tested systems resulted in higher utilization capacities. However, when charging with ±6 mA cm^–2^, the capacity utilization for systems YP-80F (26.8 vs 35.3%) and C65 (22.7 vs 32.2%) was significantly lower than that obtained from the long-term cycling stability test (Figure 3). Although in the latter test a (fast)­CC-(slow)­CV process was utilized, its CV step was limited by cutoff values of ±6 mA cm^–2^, so both procedures provide sufficient time for the boosters to be charged. Also, charging with diluted ferricyanide solutions was not a problem to access higher capacity utilization values with the CC–CV process, exhibiting the fast electron transfer kinetics for the water-soluble electroactive material. Then, the detected differences are related to the boosters.

**Table 4:** Extracted Data from Figure 4 ^a^

CC (mA/cm^2^)	Booster C65 utilization %	Booster YP-80F utilization %	Booster YP-50F utilization %
72 (cycle 9)	9.0	1.5	6.7
60	10.0	3.9	8.5
48	11.5	6.9	10.1
36	12.9	9.4	12.0
24	14.2	12.3	14.2
12	17.6	17.8	18.1
6	22.7	26.8	23.4

a Average of the cycles is considered.

For posolytes examined in this section, a similar amount of theoretical storage capacity from boosters and solution was considered, while for long-term cycling stability tests (Figure 3), boosters’ theoretical storage capacities far exceed those of the solution. Although the differences in the capacity utilization (which were expected to be practically the same) detected from both experiments are not clearly understood yet, this phenomenon was also observed by Rourke et al.^57^ The authors related this phenomenon to a mixed-limited process, because the redox mediation reaction is only occurring during a small window of the entire discharge cycle. Surprisingly, the YP-50F system maintained a similar capacity utilization with both procedures. Thus, it is likely that this carbon material has improved the electron transfer kinetics in the PW-based boosters, since it again exhibited the lowest charging–discharging time duration. Therefore, it is promising for improving the capacity utilization of the YP-50F-based system. Therefore, this system was selected to proceed to the next stage to continue cycling under a different protocol.

From Figure 4, showing the results obtained with protocol CC (fast, 72 mA cm^–2^)–CC (slow, 3 mA cm^–2^), it seems that the YP-50F system was able to take advantage of slow charging to achieve a capacity utilization value of 48.0%. To determine whether providing more time could increase this capacity, the system was cycled under a similar protocol CC (fast) - CC (slow 3 mA cm^–2^) but varying the current density at the first CC step (called as fast). The capacity utilization of the boosters as a function of cycle number is illustrated in Figure 6.

**Figure 6:**
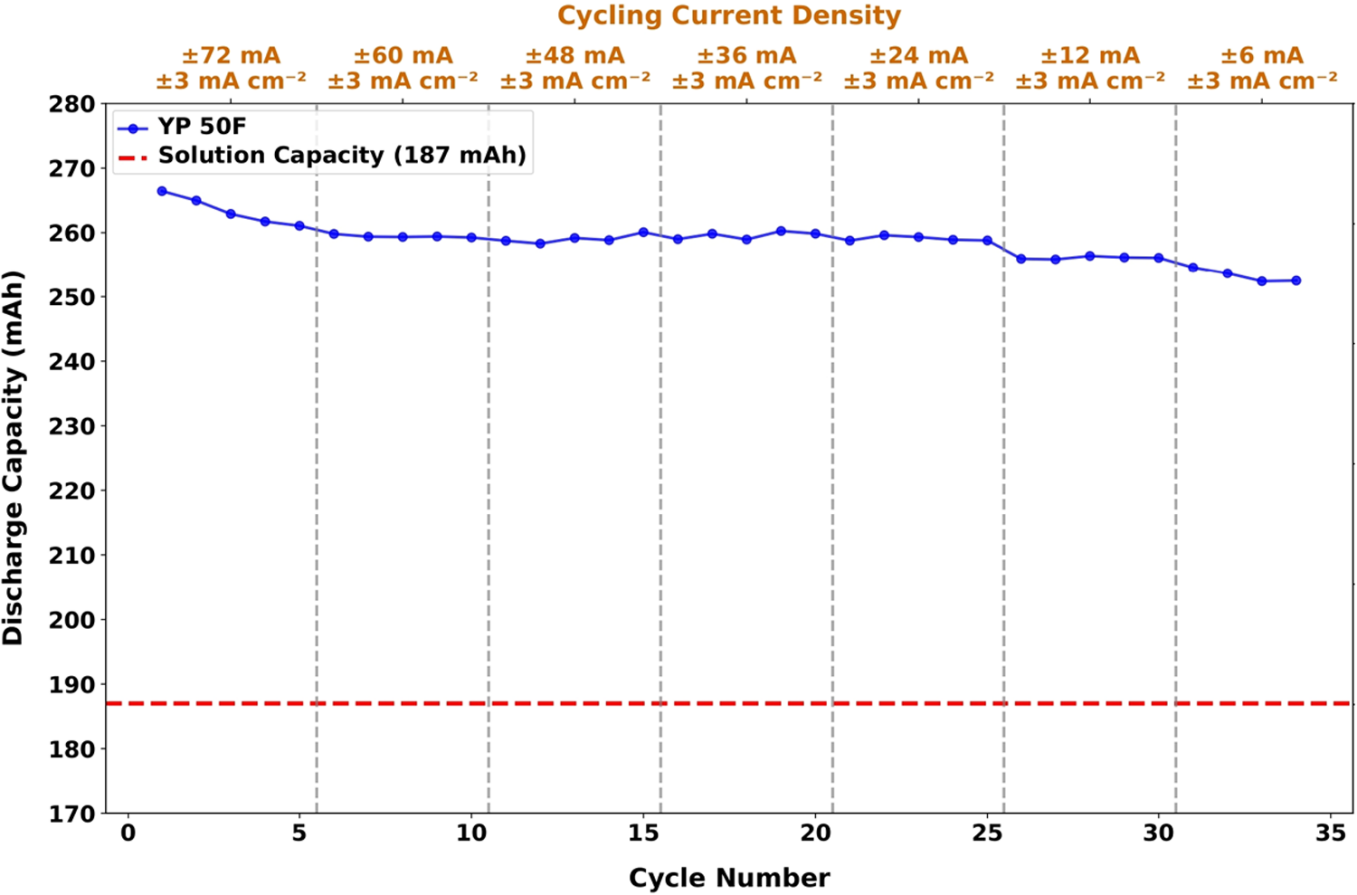
YP-50F as boosters with a capacity of 144.1 mAh in different CC (fast)-CC (slow) process conditions. 20 mL of 0.35 mol L^–1^ ferricyanide in 0.5 mol L^–1^ NaCl and 0.5 mol L^–1^ KCl solution was placed as the posolyte (187.6 mAh), incorporating boosters with 144.1 mAh capacity in the tank. 0.35 mol L^–1^ ferrocyanide + 0.035 mol L^–1^ ferricyanide in the same supporting electrolyte was placed in excess volume as the negolyte. The battery is the continuation of the test done in Figure 5.

The results obtained from Figure 6/Table 5 also show a similar capacity utilization data to that presented in Figure 4. Surprisingly, the system in Figure 6 did not show improvements when giving more time (by decreasing the first fast CC current density) for electrogenerated species to interact with the boosters, which would seem to indicate that ∼50% is the maximum capacity utilization that we can get for the boosters prepared with the carbon material YP-50F, at least under the experimental conditions tested so far.

**Table 5:** Extracted Data from Figure 5 ^a^

Current density (mA/cm^2^)	Capacity (mAh)	YP-50F booster utilization %
±72 ± 3	263.4	53.0
±60 ± 3	259.4	50.3
±48 ± 3	259.0	50.0
±36 ± 3	259.6	50.4
±24 ± 3	259.1	50.0
±12 ± 3	256.1	47.9
±6 ± 3	253.3	46.0

a Average of the cycles is considered.

By examining the testing conditions, slowly charging the cell allows more time for electrogenerated electroactive materials to interact, but this condition also restricts the SOC of the tank for a long time until enough time has passed to convert most of the ferri species to ferro. On the contrary, charging and discharging with ±72 mA cm^–2^ followed by ±3 mA cm^–2^ allowed us to quickly operate at a high SOC while giving enough time for the boosters to be charged. Therefore, we can state that high concentrations of ferro species are necessary to maximize the charging process of the boosters, as was also suggested by Ergun et al.^58^ This approach makes sense, since in this work, capacity utilizations higher than those reported by the authors with 0.2 mol L^–1^ of water-soluble electroactive species were achieved.

Finally, a comparison between the results obtained with the two utilized protocols is exemplified in Figure 7, where significant differences in charging–discharging times are visible. In the CC–CC approach, a total capacity of 250 mAh was achieved within 7 h and 22 min. In contrast, the one-step CC method delivered only 242 mAh over a considerably longer period of 32 h and 10 min. Therefore, the CC–CC protocol optimizes the testing time.

**Figure 7:**
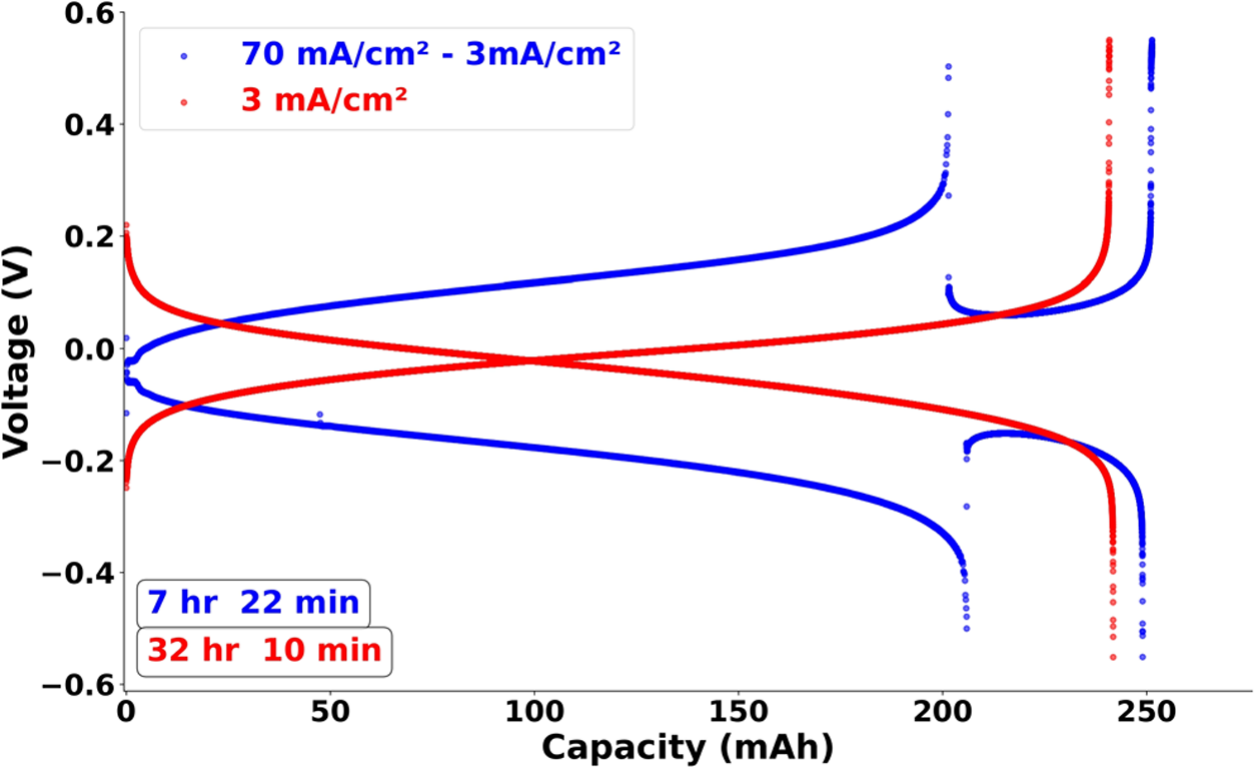
Comparison of two different processes: 70 mA/cm^2^ (fast CC) and 3 mA/cm^2^ (slow) (blue) vs 3 mA/cm^2^ (one-step CC) (red) process. 20 mL of 0.35 mol L^–1^ ferricyanide in 0.5 mol L^–1^ NaCl and 0.5 mol L^–1^ KCl was placed as the posolyte (187.6 mAh), including boosters with 144.1 mAh capacity. 0.35 mol L^–1^ ferrocyanide +0.035 mol L^–1^ ferricyanide was placed in excess volume in the same electrolyte as the negolyte.

### Electrolyte Effects on the Boosters’ Capacity Utilization

In the previous section, the effect of the carbon material on the flow battery performance of PW-based boosters was discussed. A significant effect of the water-soluble electroactive material on the performance of the boosters was also examined previously in KCl solutions, limiting the study to low concentrations of this electroactive material.^57^ For the systems studied here, electrolyte compositional effects were also examined in batteries containing a higher concentration (0.8 mol L^–1^) of K_3_ [Fe­(CN)_6_] in 1 mol L^–1^ NaCl. Larger battery cells (20 and 9 cm^2^ membrane area) were utilized to cycle the solution without significantly increasing the charging–discharging times. The tests were done with a CC–CC process applying ±37.5 mA cm^–2^ and then 2.5 mA cm^–2^, successively. As can be seen in Figure 8, three different carbon materials were utilized in the preparation of the booster. For YP-50F-based boosters, the capacity utilization resulted to be 94.8% and this capacity remained stable for 56 days of testing. On the other hand, a stable 76.7% capacity utilization (over 100 cycles) was achieved with boosters containing the carbon materials YP-80F and BP2000. The flow battery cell for these cases was bigger, using a higher concentration of electrolyte, so the time for each cycle was longer. The BP2000 system again experienced material disintegration after 28 days of cycling, so the mechanical stability was not affected by the concentration of ferricyanide or the electrolyte (Figure S5). The tests for systems YP-80F and YP-50F were aborted at cycles 117 and 72, respectively.

**Figure 8:**
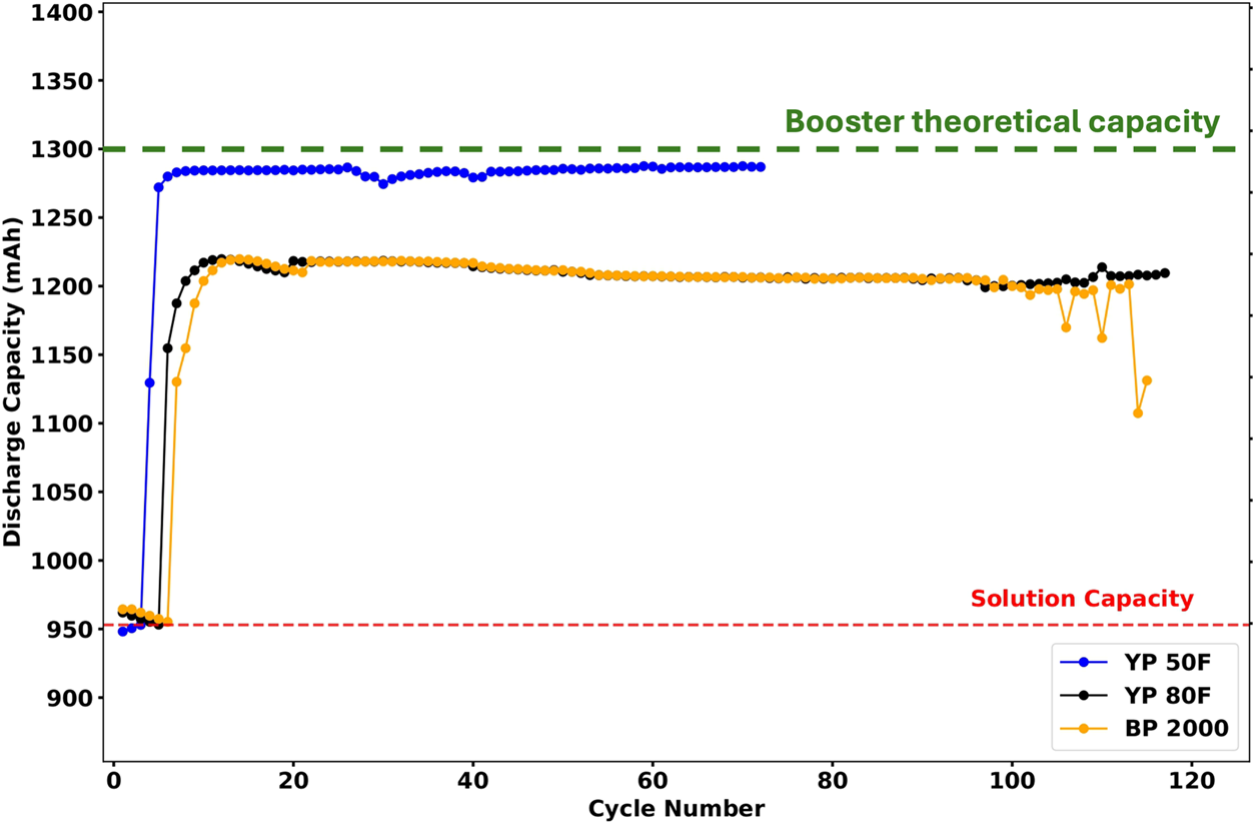
Boosters made with YP-80F, YP-50F, and BP2000 in high concentration electrolyte. Posolyte contains 50 mL 0.8 mol L^–1^ of K_3_ [Fe­(CN)_6_] in 1 mol L^–1^ NaCl + 0.01291 mol PW in each booster (346.4 mAh capacity from booster). Negolyte contains 200 mL 0.5 mol L^–1^ K_4_ [Fe­(CN)_6_] and 0.03 mol L^–1^ K_3_ [Fe­(CN)_6_] in 1 mol L^–1^ NaCl. Booster BP2000 tested in a 20 cm^2^ membrane area. YP-50F and YP-80F were tested in the cell with a 9 cm^2^ membrane area, a flow rate of 35 mL/min for all the batteries. CC–CC process applied ±37.5 mA cm^–2^ and then 2.5 mA cm^–2^ with ±0.55 V cutoff.

To shed light on the improved capacity detected, the morphology and surface chemistry of the C65 booster were examined before and after 8 days of cycling (the boosters showed minimal disintegration) by SEM (Figure 9) and Raman spectroscopy (Figure 10). SEM images a–c show the morphology of booster C65 before cycling. At lower magnifications (a,b), the booster exhibits a relatively intact and continuous surface structure, with the active material well integrated within the surrounding matrix. The higher-magnification image (c) confirms that the particle surfaces are uniformly coated with a carbon material, indicating good electronic connectivity throughout the electrode before any electrochemical cycling takes place. Following 8 days of cycling, Figure 9d,e reveals notable morphological changes despite the absence of booster dissolution, which is consistent with the electrochemical data showing capacity degradation without catastrophic material loss. Fragmented particles with irregular, platelike morphology are observed scattered across the electrode surface, visibly detached from the carbon matrix. The loss of intimate contact between these particles and the conductive carbon network is significant, as electronic connectivity is a prerequisite for electrochemical utilization. The detachment of these particles therefore provides a plausible mechanistic explanation for the observed capacity fade, as disconnected fragments would no longer actively participate in the charge-storage process regardless of their chemical integrity. Since the four boosters under comparison differ only in their carbon type while sharing the same binder composition, the intrinsic morphology of each carbon is expected to play a significant role in determining the structural stability and electrochemical performance of the electrode. A more favorable carbon morphology can promote better particle interconnectivity and a more robust electrode architecture.

**Figure 9:**
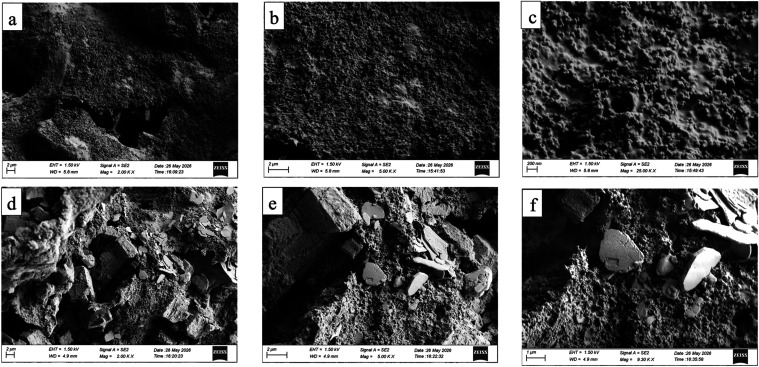
SEM images of booster C65 (a–c) before cycling (d–f) after 8 days cycling with capacity loss, in a tank containing 0.2 mol L^–1^ ferrocyanide and 1 mol L^–1^ KCl.

**Figure 10:**
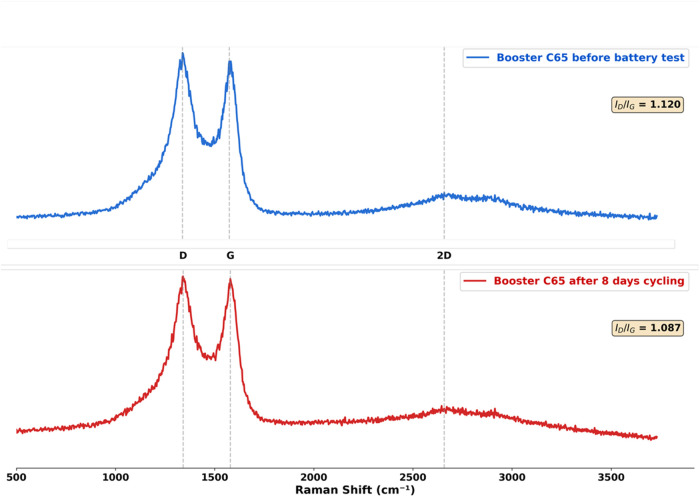
Raman spectra of booster C65 before cycling (blue) and after 8 days of cycling (red).

Raman spectra (Figure 10) for both samples before and after cycling show the characteristic D band (∼1350 cm^–1^), G band (∼1580 cm^–1^), and 2D band (∼2700 cm^–1^) of carbon black. The *I*_D_/*I*_G_ ratio decreases marginally from 1.120 before cycling to 1.087 after 8 days of cycling, though both values remain well above 1.0, confirming that C65 retains its predominantly disordered carbon character throughout. This change is small and may indicate that cycling does not significantly alter the carbon framework of the C65 booster by 8 days of cycling.

Concentration effects on the performance of PB-based boosters were previously examined using low concentrations of ferricyanide species up to 0.2 mol L^–1^.^58^ As a higher concentration of K_4_ [Fe­(CN)_6_] was not investigated, the maximum boosters’ capacity utilization was 35%, which is too low. In the other work,^57^ by trying different carbon materials at 0.5 mol L^–1^ of K_4_ [Fe­(CN)_6_], the authors reached a maximum capacity utilization of 46%. With this information, it remains unclear whether the composition of the boosters or the concentration of the solution has a greater impact on determining the maximum capacity utilization. In this work, regardless of the carbon material used in the formulation with PW, increasing solubility of the water-soluble electroactive material with a mixture of ions K^+^ and Na^+^, allowed us to demonstrate that higher concentration of ferricyanide in the electrolyte is crucial to significantly improve in boosters’ capacity utilization, reaching values close to those ones reported by pioneering works for PB-based boosters: 57.6% and 61.0%, by Chen et al. (0.8 mol L^–1^ K_3_Fe­(CN)_6_ in 1 mol L^–1^ KCl)^59^ and Yan et al. (0.3 M K_4_Fe­(CN)_6_ + 1.0 M KCl),^38^ respectively. The values obtained are also comparable to those reported (ranging from 67% to 83%) by Marin-Tajadura et al.,^70^ although in their work, the capacity enhancement was primarily driven by the monolithic booster design, which maximizes flow distribution across the reservoir. For the PW-based boosters tested here, the CC­(fast)-CC­(slow) charging–discharging protocol was also crucial to reach maximum utilization capacities.

As increasing ferricyanide in solution indirectly incorporates more K^+^ species into the solution, the next step was to examine which of the components in the charge-transporting solution is related to enhancing the capacity utilization of the boosters. For this, we utilized cyclic voltammetry to examine the electrolyte effects on the electrochemistry of electroactive materials.

Changing K_3_[Fe­(CN)_6_] concentration in the electrochemical cell does not significantly affect the CV response (Figure 11) and the formal potential of the system. The potential shifts were also marginal when mixing counterions, as illustrated in Figure S6. Therefore, notable improvements in boosters’ performance cannot be explained by better potential matching resulting from potential shifts of ferrocyanide.

**Figure 11:**
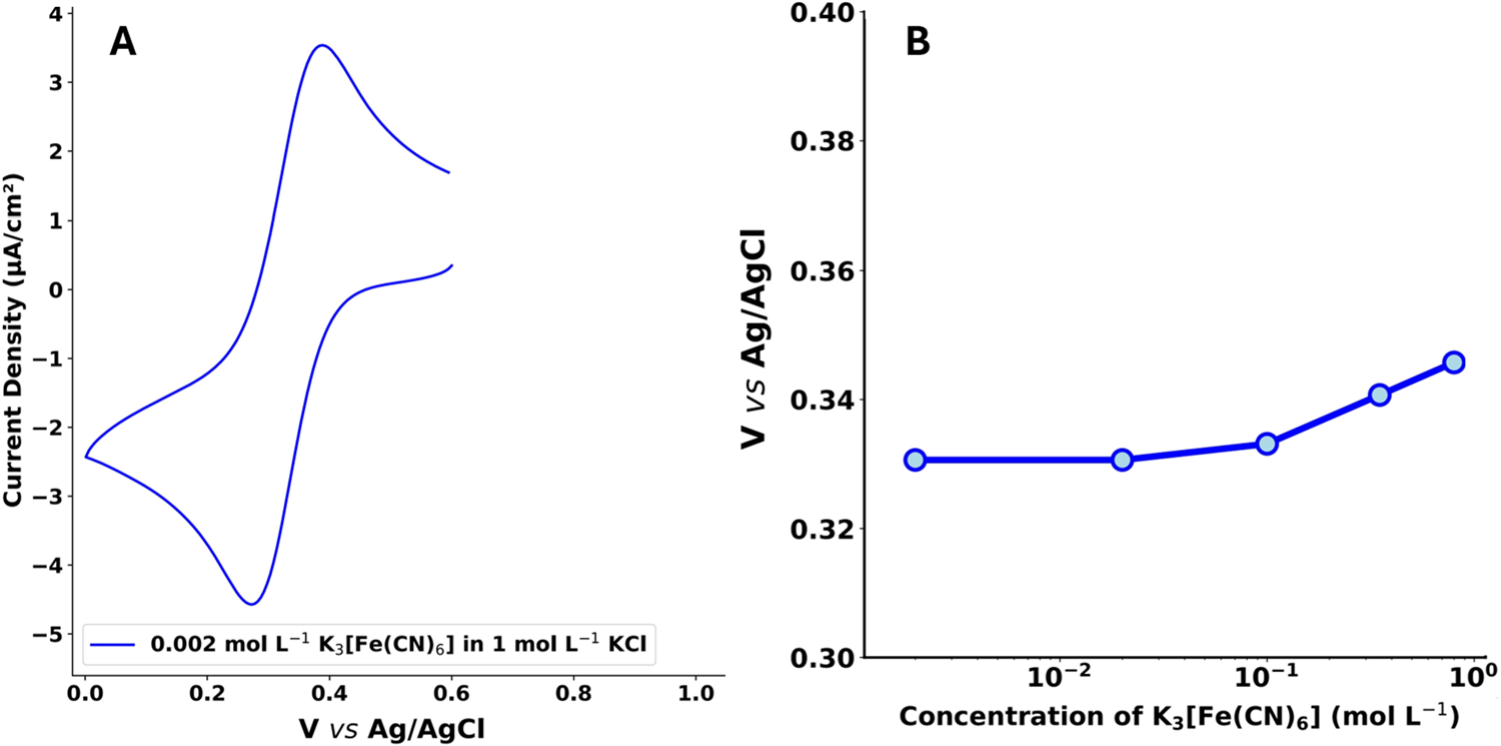
Cyclic voltammogram of K_3_[Fe­(CN)_6_] at 0.002 mol L^–1^. (A) potential shift of K_3_[Fe­(CN)_6_] from 0.002 to 0.8 mol L^–1^ in 1 mol L^–1^ KCl vs Ag/AgCl (3 mol L^–1^ KCl) reference electrode. (B) Scan rate 100 mV s^–1^.

Because of solubility issues, cyclic voltammetry for PB/PW was performed on the soluble fraction of a low-concentration solution. The CV (Figure 12) wave does not significantly change its shape, but its redox potential shifts to positive values with increasing KCl in solution. This phenomenon may be partially related to the capacity changes observed in cycled boosters.

**Figure 12:**
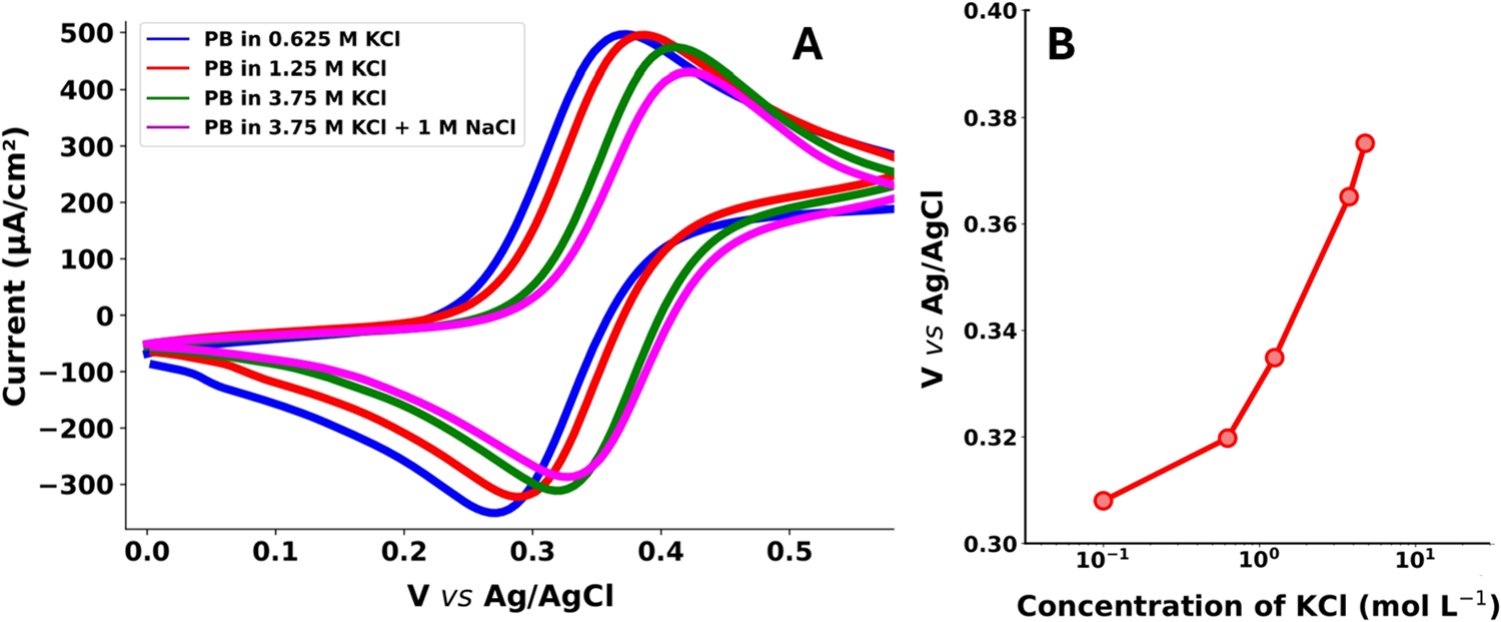
Cyclic voltammograms of PB/PW in different KCl concentrations from 0.625 mol L^–1^ to 3.75 mol L^–1^ vs Ag/AgCl (3 mol L^–1^ KCl) reference electrode. Scan rate 10 mV s^–1^, (A) potential shift of Prussian White by increasing the KCl concentration (B).

By increasing the electrolyte concentration, the availability of K^+^/Na^+^ ions within the booster beads increases, which may also have kinetic effects. In addition, the indirect introduction of K^+^ species through a higher ferrocyanide concentration may influence the thermodynamics of the PB/PW redox couple, potentially contributing to a better redox matching between the water-soluble and insoluble electroactive species in the liquid and solid phases, respectively. As the changes in the redox matching are rather small, it is proposed that the improvements in the booster utilization with increasing concentrations are due to improved mass transfer between solids, especially inside the beads and liquid. By increasing the electrolyte concentration, the flux of available ferri-/ferrocyanide and K^+^/Na^+^ ions into the booster beads increases, accelerating mass transfer and enhancing intercalation kinetics. While ionic matching effects may contribute to some extent, the primary driver of the observed performance improvement is likely the higher ion availability at the solid electrolyte interface.

As the wave shape remains unchanged at all KCl concentrations utilized, the global process behaves as reversible with fast charge-transfer kinetics, where the potential shifts observed follow the Nernst equation described previously, which accounts for the dependence of the redox potential on K^+^ intercalation into the PB/PW lattice.^71,72^ Therefore, for future work, finding a proper concentration and mixture ratio of species K^+^ and Na^+^ in the charge-transporting electrolyte will be crucial to maintain a good K_3_[Fe­(CN)_6_] water solubility, while maximizing the capacity utilization of the boosters through a better redox matching.

## Conclusion

To shed light on how different system components influence the performance of Prussian White-based boosters in flow batteries, boosters prepared with four commercially available carbon materials (at 10% of the total composition) were cycled in symmetric cells with ferri/ferrocyanide-based electrolytes of distinct compositions.

A 21–22 day charge–discharge cell test was applied to all batches of boosters obtained to demonstrate their long-term cycling stability. Boosters containing the carbon material YP-50F underwent at least twice as many charge–discharge cell cycles in this test compared to the remaining systems studied (e.g., 500 vs 194, for YP-50F and C65, respectively), exhibiting faster charge–discharge behavior at lower capacity utilization.

Variations of the carbon material in the formulation of the boosters produced systems with capacity utilizations ranging from 22% to 36% at low ferrocyanide concentrations (0.1 mol L^–1^). A significant improvement in this capacity was obtained when charging and discharging the boosters with high concentrations (0.8 mol L^–1^) of ferrocyanide, revealing a critical role of the electrolyte composition in enhancing the capacity utilization of the boosters. Under this condition, a maximum capacity utilization of 94.8% was obtained for the system containing the carbon material YP-50F.

Cyclic voltammetry revealed that this phenomenon is related to changes in the thermodynamics of the PB/PW redox couple, which is influenced by the addition of K^+^ ions to the medium when potassium ferri-/ferrocyanide is added to the electrolyte. In general, it is proposed that increasing the latter species generates an improved mass transfer between the solids, especially inside the beads and the ions in the liquid. Together, these results indicate that booster capacity utilization can be improved through the selection of appropriate carbon materials and an increased mediator concentration.

## Supplementary Material



## Notes

Electrochemical characterizations (cyclic voltammograms and a battery test) and photos of some cycled batteries (PDF)
