# Supplementary material for: Carbon Material and Electrolyte Composition Effects on the Performance of Prussian White Solid Boosters in Flow Batteries
Source: ACS Omega. 2026 Jul 24;11(31):46554–67. doi: 10.1021/acsomega.6c04178 (PMC13470904; doi:10.1021/acsomega.6c04178)
Supplement: Supplementary file 1 [file ao6c04178_si_001.pdf]

SI

Carbon material and electrolyte composition effects on the performance of Prussian White solid boosters in flow batteries

Vahid Abbasi [//] [a,b]

Eduardo Martínez González [//] [a,b*]

Rosa Tirronen [b]

Ayesha Kousar [a]

Eeva-Leena Rautama [a]

Pekka Peljo [a,b*]

a) Department of Chemistry and Materials Science Aalto University, 00076 Aalto, Finland

b) Mechanical and Materials Engineering Department University of Turku, 20014 Turku, Finland

edumg109@gmail.com, eduardo.martinezgonzalez@aalto.fi; pekka.peljo@aalto.fi

KEYWORDS: flow battery, solid boosters, Prussian White, capacity utilization, carbon effects, electrolyte effects, counterion effects

Table of Contents:

Figure S1. SEM images for a) BP2000 b) C65 c) YP-50F and d) YP-80F. Reproduced from ref [1] under license number: 6280840049589, ref [2] under license number 6281871209667, and ref [3] under license CC BY 4.0

Figure S2. Raman spectra of four carbon black materials (YP-80F, YP-50F, Super C65, and BP2000) showing the D band (~1350 cm^{-1}), G band (~1580 cm^{-1}), and 2D band (~2700 cm^{-1}). Reproduced from ref [4] under license CC BY 3.0, ref [3] under license CC BY 4.0, ref [5] under license 6281880587087, and ref [6] under license CC BY 4.0

Figure S3. Pore width of four different carbon used. Reproduced from ref [1] under license number: 6280840049589, ref [4] under license CC BY 3.0, ref [7] under license CC BY 4.0, and ref [6] under license CC BY 4.0

Figure S4. Setup for performing batteries, boosters (YP-50F type here) are locked by glass wools in the middle of the solution

Figure S5. Booster made with carbon material BP 2000 after breaking down to smaller pieces over cycling

Figure S6. 0.35 mol L^{-1} $K_3[Fe(CN)_6]$ in 1 mol L^{-1} KCl (red) and 0.5 mol $^{-1}$ KCl + 0.5 mol L^{-1} NaCl solution (blue) (left), and 0.8 mol L^{-1} $K_3[Fe(CN)_6]$in 1 mol L^{-1} KCl (purple) and 1 mol L^{-1} NaCl (green) (right) all potentials *vs* Ag/AgCl (3 mol L^{-1} KCl) reference electrode. Scan rate 100 mVs^{-1}

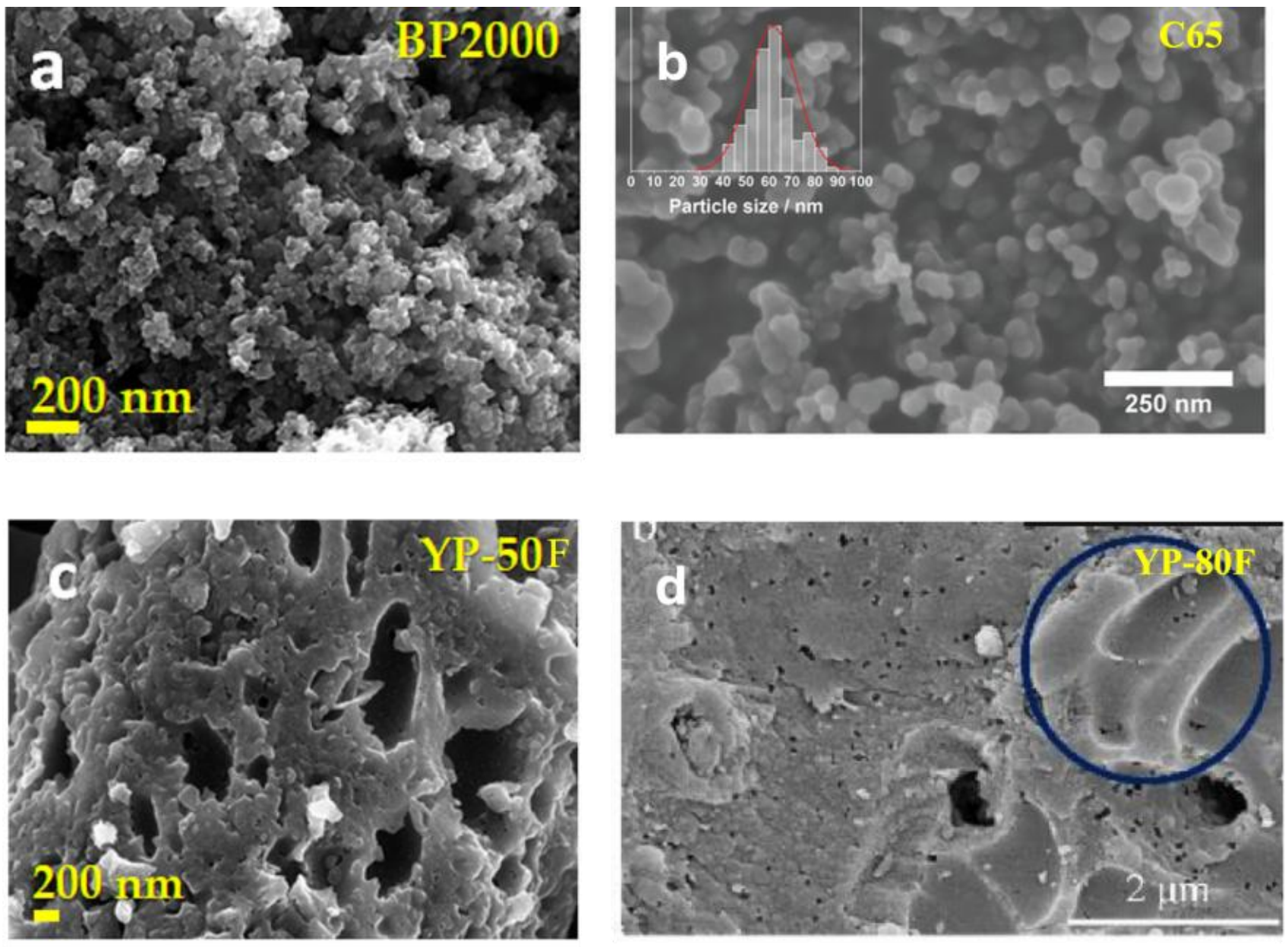

Figure S1. SEM images for a) BP2000 b) C65 c) YP-50F and d) YP-80F. Reproduced from ref [1] under license number: 6280840049589, ref [2] under license number 6281871209667, and ref [3] under license CC BY 4.0

SEM images of the four carbon materials reveal distinct morphological differences that are relevant to electrode stability and performance. BP2000 (a) presents densely aggregated nanoparticles in the 20–50 nm range, forming an open and interconnected network with large interparticle voids. Super C65 (b) consists of spherical primary particles of approximately 60 nm that aggregate loosely, with porosity predominantly located between particles. Both materials share a structurally important characteristic: the carbon exists as discrete nanoscale particles held together primarily by physical contact forces rather than by any rigid framework. The interparticle pore volume in both cases is open and macroporous, allowing electrolyte to penetrate freely and deeply into the aggregate structure.

YP-50F (c) and YP-80F (d), by contrast, consist of continuous monolithic activated carbon particles in which the porosity is contained within the particle interior rather than existing as voids between separate units. The particle itself forms a rigid, covalently bonded carbon matrix, which represents a fundamentally different structural arrangement compared to the nanoparticulate carbons above.

These morphological distinctions are likely to influence how well each carbon maintains structural integrity and electronic connectivity within the electrode over extended cycling.

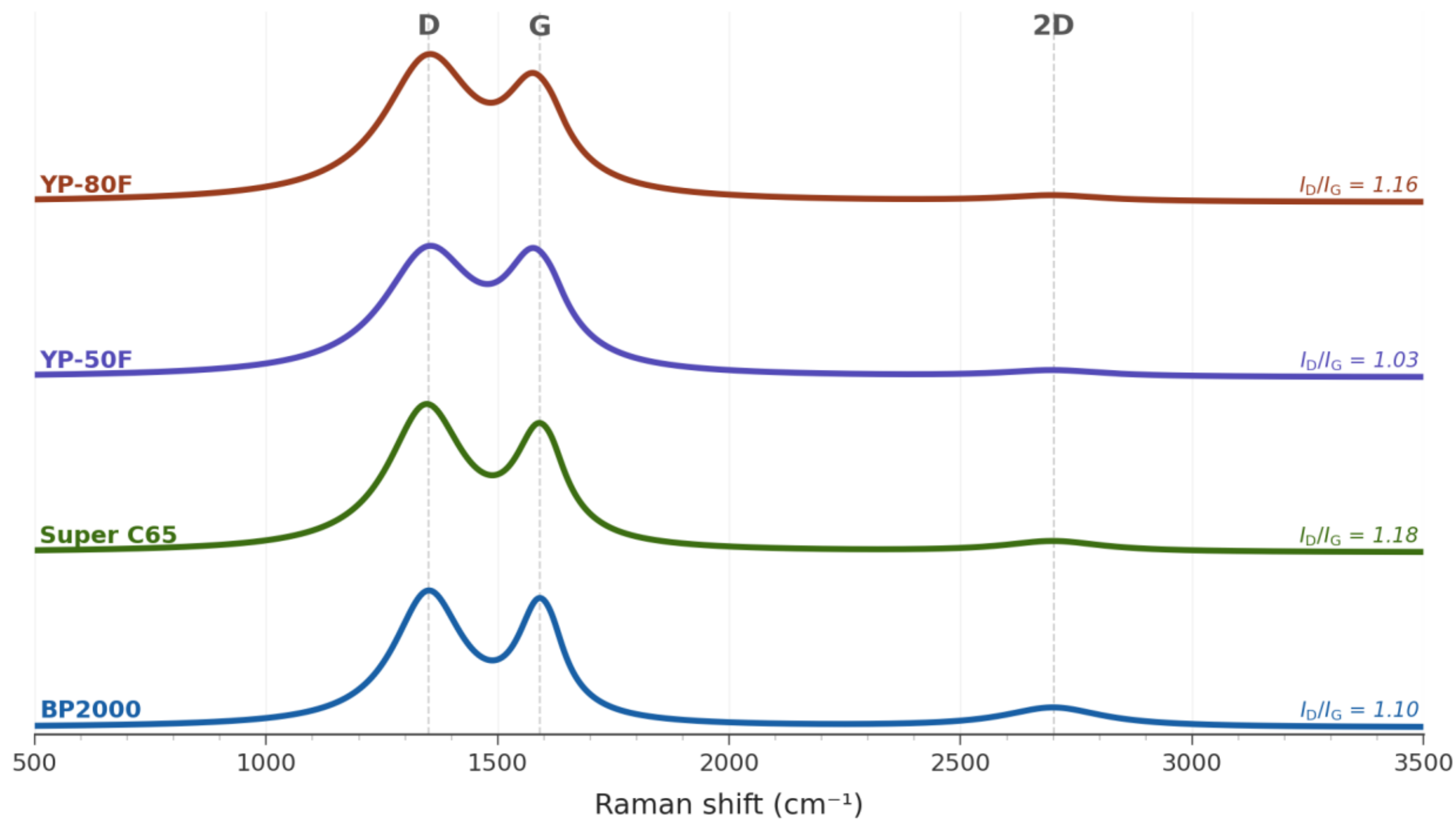

Figure S2. Raman spectra of four carbon black materials (YP-80F, YP-50F, Super C65, and BP2000) showing the D band (~1350 cm^{-1}), G band (~1580 cm^{-1}), and 2D band (~2700 cm^{-1}). Reproduced from ref [4] under license CC BY 3.0, ref [3] under license CC BY 4.0, ref [5] under license 6281880587087, and ref [6] under license CC BY 4.0

All four carbon blacks show Raman spectra typical of highly disordered sp^2 carbon, with broad, heavily overlapping D and G bands centered near 1350 and 1580 cm^{-1} respectively, and a weak, broad 2D band around 2700 cm^{-1} that reflects the near-absence of long-range graphitic order. The I_D/I_G ratios range narrowly from 1.03 (YP-50F) to 1.18 (Super C65), indicating that all four materials have a similar degree of structural disorder, with no single sample standing out as markedly more graphitic than the others.

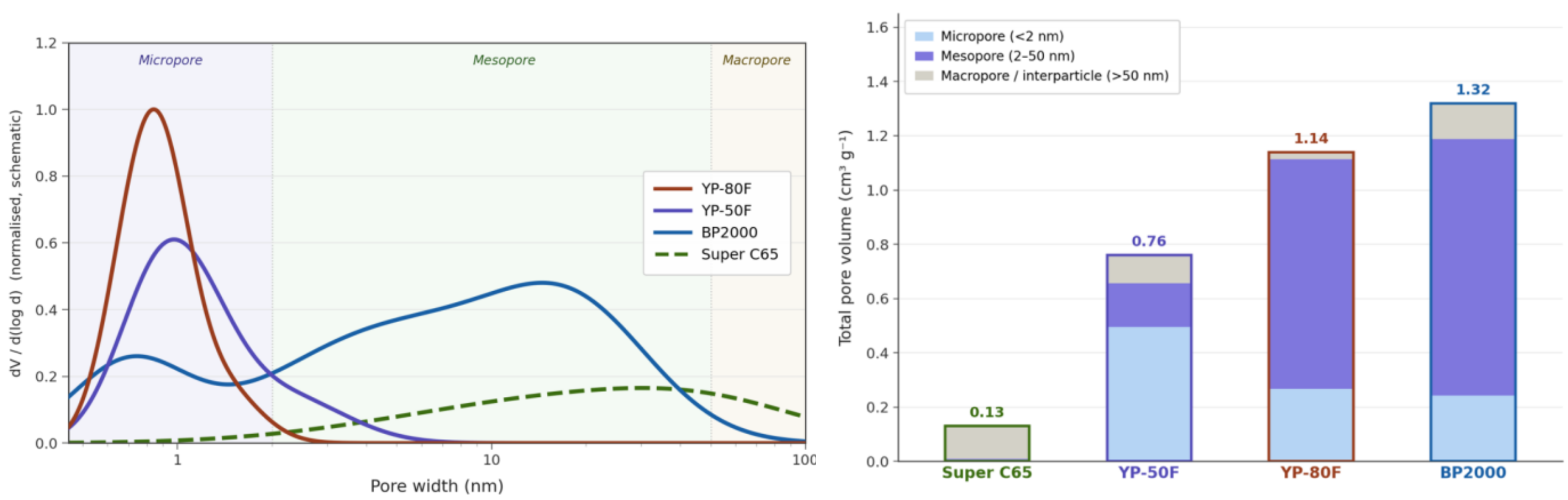

Figure S3. Pore width of four different carbon used. Reproduced from ref [1] under license number: 6280840049589, ref [4] under license CC BY 3.0, ref [7] under license CC BY 4.0, and ref [6] under license CC BY 4.0

Picture of the setup is shown as Figure S4. The posolyte tank keep the booster particles in the middle and locked by glass wool to prevent being washed to the cell by flow and block the flow.

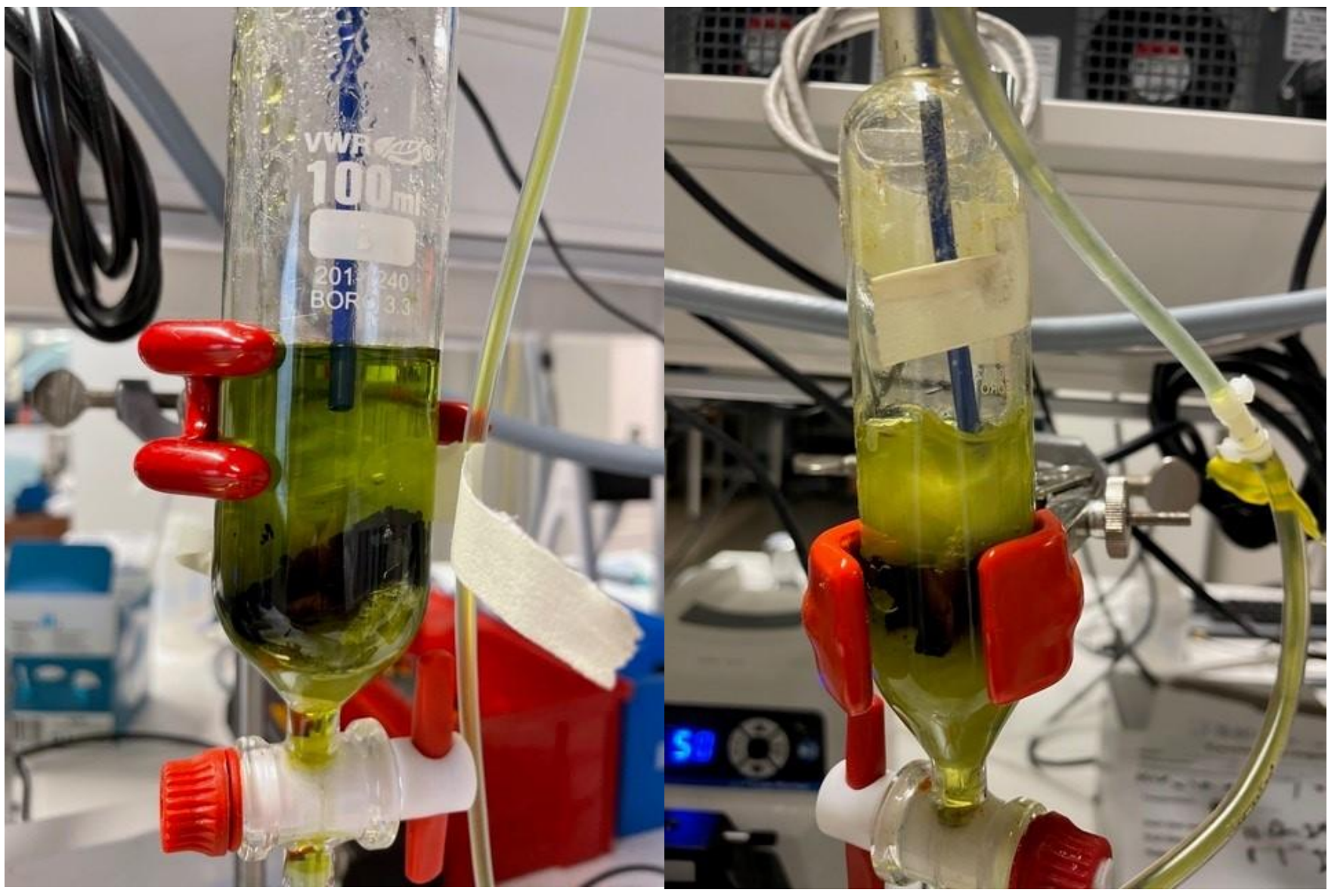

Figure S4. Setup for performing batteries, boosters (YP-50F type here) are locked by glass wools in the middle of the solution

In Figure S5, the posolyte tank from the booster made with carbon material BP2000 is shown. When it dissolved after cycling, even the glass wool cannot keep them, and the particles breaks down to smaller pieces that they block the flow in the cell.

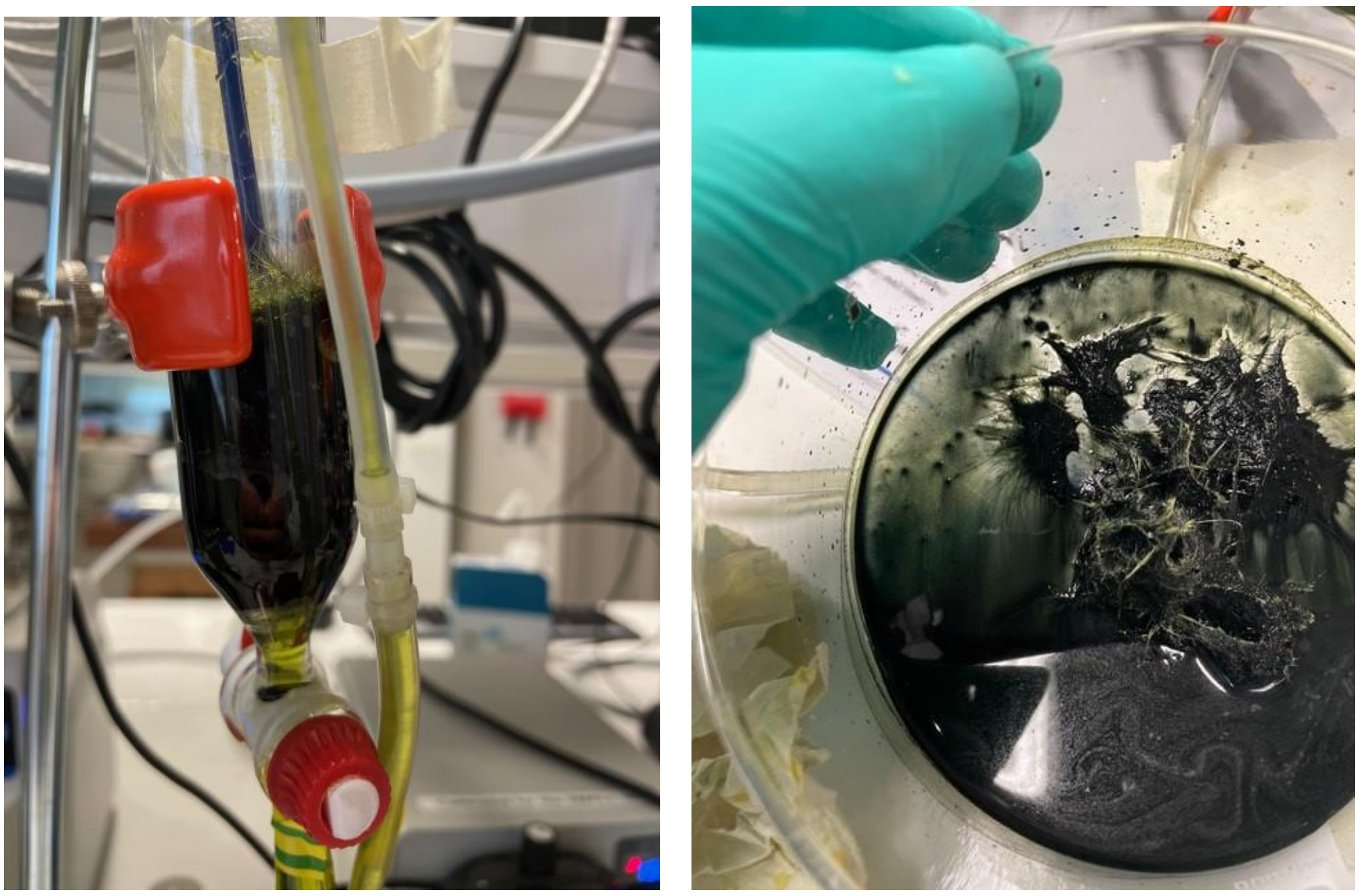

Figure S5. Booster made with carbon material BP 2000 after breaking down to smaller pieces over cycling

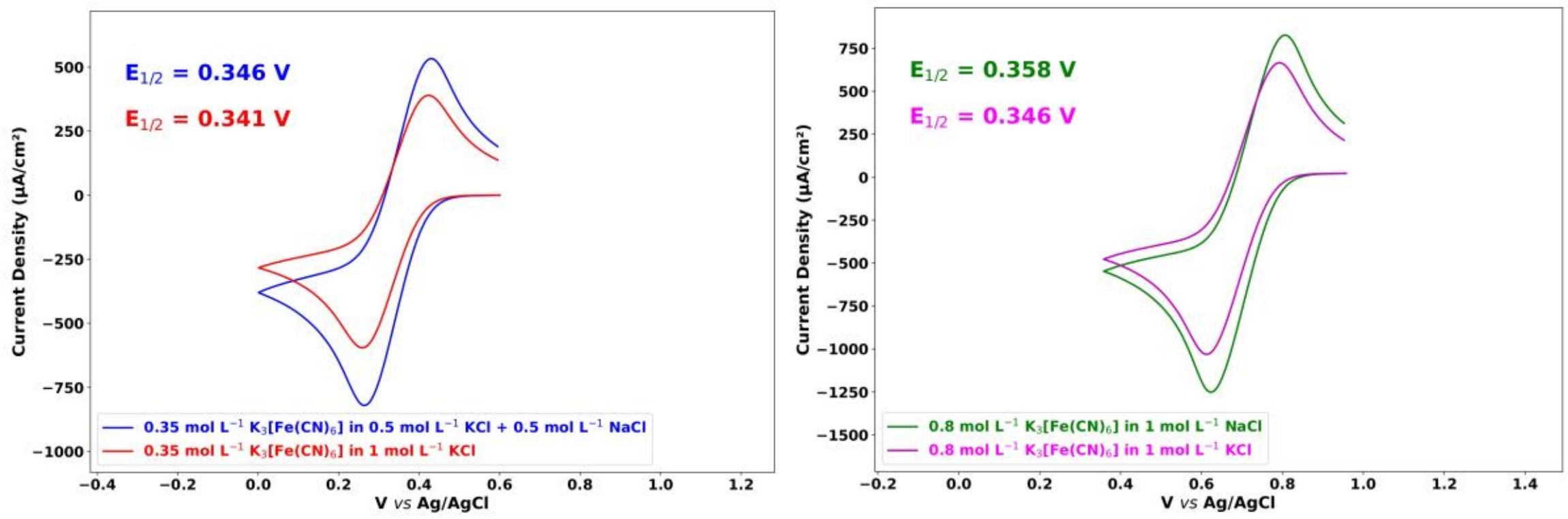

Figure S6. 0.35 mol L^{-1} $K_3[Fe(CN)_6]$ in 1 mol L^{-1} KCl (red) and 0.5 mol $^{-1}$ KCl + 0.5 mol L^{-1} NaCl solution (blue) (left), and 0.8 mol L^{-1} $K_3[Fe(CN)_6]$in 1 mol L^{-1} KCl (purple) and 1 mol L^{-1} NaCl (green) (right) all potentials *vs* Ag/AgCl (3 mol L^{-1} KCl) reference electrode. Scan rate 100 mVs^{-1}

References:

1) Yu, G. T.; Hsieh, T. H.; Lin, Y. H.; Lin, J. H. Porous Carbon Nanosheets Derived from Spent Coffee Grounds for Highly Stable Electric Double-Layer Capacitors Enabled by Structurally Complementary Carbons. *ACS Sustainable Resource Management* **2024**, *1* (1), 76–87. https://doi.org/10.1021/acssusresmgt.3c00039.

2) Zhang, K.; Sun, J.; E, L.; Ma, C.; Luo, S.; Wu, Z.; Li, W.; Liu, S. Effects of the Pore Structure of Commercial Activated Carbon on the Electrochemical Performance of Supercapacitors. *J. Energy Storage* **2022**, *45*. https://doi.org/10.1016/j.est.2021.103457.

3) Tengen, B.; Winkelmann, T.; Ortlieb, N.; Perner, V.; Studer, G.; Winter, M.; Esser, B.; Fischer, A.; Bieker, P. Immobilizing Poly(Vinylphenothiazine) in Ketjenblack-Based Electrodes to Access Its Full Specific Capacity as Battery Electrode Material. *Adv. Funct. Mater.* **2023**, *33* (9). https://doi.org/10.1002/adfm.202210512.

4) Weingarth, D.; Drumm, R.; Foelske-Schmitz, A.; Kötz, R.; Presser, V. An Electrochemical in Situ Study of Freezing and Thawing of Ionic Liquids in Carbon Nanopores. *Physical Chemistry Chemical Physics* **2014**, *16* (39), 21219–21224. https://doi.org/10.1039/c4cp02727b.

5) Tang, Y.; Lu, L.; Zang, X.; Wang, B.; Ye, X. Tailored Properties of Carbon for Supercapacitors by Blending Lignin and Cellulose to Mimic Biomass as Carbonaceous Precursor. *ChemSusChem* **2023**, *16* (17). https://doi.org/10.1002/cssc.202300357.

6) Xu, Z.; Pedersen, A.; Shimizu, S.; Yoshii, T.; Nishihara, H.; Titirici, M.-M.; Barrio, J.; Forse, A. C. Exploring Improved Supercapacitor Electrodes for Electrochemical Carbon Dioxide Capture. *ACS Electrochemistry* **2026**, *2* (5), 1115–1120. https://doi.org/10.1021/acselectrochem.6c00086.

7) Yu, J.; Wang, Z.-L.; Ma, T. Tuning Surface Molecular Design of Porous Carbon for Blue Energy Harvesting. *Research* **2023**, *6*. https://doi.org/10.34133/research.0173.
